# Digital media exposure and pediatric health: the recommendations from the Italian Society of Pediatrics Digital Dependency Commission

**DOI:** 10.1186/s13052-026-02198-6

**Published:** 2026-01-23

**Authors:** Elena Bozzola, Mariangela Irrera, Sarah Barni, Cinthia Caruso, Bianca Leccese, Enrica Franzese, Lavinia Fioretti, Luca Bernardelli, Elena Scarpato, Vita Cupertino, Teresa Mazzone, Rocco Russo, Alfonso Benevento, Barbara Strappato, Marco Cervellini, Pietro Ferrara, Rino Agostiniani

**Affiliations:** 1 Bambino Gesù Children’s Hospital, IRCCS, Rome, Italy; 2 The Italian Pediatric Society Digital Dependency Commission, Rome, Italy; 3 University Tor Vergata, Rome, Italy

**Keywords:** Media device, Social media, Children, Adolescent, Pediatric health

## Abstract

**Background:**

Digital media exposure has become an integral component of childhood and adolescence, profoundly reshaping developmental environments worldwide. The Italian Pediatric Society (SIP) Digital Dependency Commission aim to provide indications for families and paediatricians to minimize the potential consequences related to media device and social media use in minors.

**Materials and methods:**

A systematic review of the international literature was performed in Pubmed searching for Mesh key terms from 1st January 2018 to 1st April 2025. Outcomes of interest encompassed physical health, obesity, neurocognitive development, sleep, visual health, mental well-being, behavioural addiction, and cyberviolence.

**Results:**

The analysis demonstrates that excessive and unregulated digital media exposure is associated with sedentary behaviour, unhealthy dietary patterns, obesity, and cardiometabolic risk. High screen use is linked to language delays, impaired attention, reduced executive functioning, and structural brain changes. Evidence reveals sleep disruption, with delayed sleep onset, reduced duration, and circadian rhythm disturbances. Visual health is compromised through increased symptoms of digital eye strain and accelerating myopia progression. Mental health outcomes include anxiety, depressive symptoms, emotional dysregulation, loneliness, and social comparison stress. Problematic digital, gaming disorder, cyberbullying and online violence often coexist with psychological distress and emotional and behavioural difficulties.

**Discussion:**

The findings highlight digital media exposure as a multifaceted and powerful determinant of pediatric health. On the base of these findings, the SIP Digital Dependency Commission provide the following recommendations:

- Delay introduction of personal smartphones until at least 13 years of age; use simplified models until 18.
- Avoid unsupervised Internet access before age 13; enable parental controls thereafter.
- Postpone social media access ideally until 18, but not earlier than 14 years.
- Prohibit device use during meals, before bedtime, and within bedrooms.
- Encourage outdoor activities, sports, reading, and creative play as primary developmental experiences.
- Promote school-based digital literacy education, including privacy protection, cyber-ethics, and critical evaluation of sources.
- Pediatricians should routinely evaluate screen habits during visits, screen for risk factors, and counsel families using anticipatory guidance.

**Conclusion:**

Coordinated action by clinicians, educators, parents, and policymakers is essential to promote a balanced and healthy digital ecosystem for children and adolescents.

**Supplementary Information:**

The online version contains supplementary material available at 10.1186/s13052-026-02198-6.

## Background

Over the last two decades, the rapid proliferation of digital devices has reshaped the behavioral, social, and developmental landscape of children and adolescents. Smartphones, tablets, laptops, and connected toys are now ubiquitous, providing unparalleled access to information, communication, and entertainment. However, the health impact of this technological integration into daily life has raised growing concern among pediatricians and child health researchers. A large body of evidence now suggests that prolonged exposure to screens and online environments during developmental years may interfere with physical, cognitive, and emotional maturation [1–3].

The pediatric population represents a uniquely vulnerable group because the human brain continues to develop throughout childhood and adolescence, undergoing complex neurobiological maturation of cortical and subcortical circuits. Excessive digital stimulation during this critical period may induce neuroplastic changes with long-term consequences for attention, learning, emotional regulation, and impulse control [4, 5]. Furthermore, the displacement of sleep, physical activity, and in-person social interaction can exacerbate risks for obesity, myopia, anxiety, depression, and social withdrawal syndromes such as hikikomori [6–8].

During the COVID-19 pandemic, the digital environment became an essential interface for schooling and social connection, yet it simultaneously intensified exposure to screen-related risks. In Italy and internationally, mean daily screen time among minors increased by approximately 4–6 h, doubling pre-pandemic levels [9]. This increase occurred across age groups and socioeconomic contexts, amplifying existing vulnerabilities [10].

In response to the observed rise in Internet Gaming Disorder (IGD), problematic smartphone use (PSMU), social media overexposure, and associated behavioral and health issues documented in recent epidemiological reports.

The Italian Society of Pediatrics (SIP), through its Digital Dependency Commission, aimed to synthesize current evidence and formulate practical recommendations for pediatricians, educators, and parents to foster a balanced and health-promoting approach to digital engagement. It was decided to conduct an evaluation of the available literature on digital media exposure in children and adolescents [11–13].

This manuscript presents the Commission’s integrated findings, structured by health domains: obesity, cognitive development, sleep, visual health, mental health, addiction and problematic use, cyberbullying and online violence—culminating in a set of recommendations tailored for pediatric clinical practice.

## Materials and methods

The SIP Digital Dependency Commission adopted a systematic review methodology in accordance with PRISMA guidelines. The search strategy was developed to capture peer-reviewed studies addressing behavioral, cognitive, physical, and psychosocial consequences of digital media exposure in individuals aged 0–18 years from 1st January 2018 to 1st April 2025.

Searches were performed on PubMed using the following Boolean string: ((((“Smartphone”[Mesh]) OR “Mobile Applications”[Mesh]) OR “Internet Addiction Disorder”[Mesh]) OR “Computers, Handheld”[Mesh]) OR “Computers”[Mesh]) AND (“Behavior, Addictive”[Mesh] OR “Technology Addiction”[Mesh] OR “Health Risk Behaviors”[Mesh] OR “Risk Assessment”[Mesh] OR “Risk Factors”[Mesh]). A secondary search using the MeSH term “media device” was also conducted. The inclusion criteria were: (1) studies focusing on pediatric populations (≤ 18 years) (2), quantitative or qualitative assessment of digital media exposure (3), outcomes relating to physical, psychological, or cognitive health, and (4) peer-reviewed publication in English or Italian. Exclusion criteria included non-original papers without empirical data, adult samples, or studies with insufficient methodological detail.

**Fig. 1:**
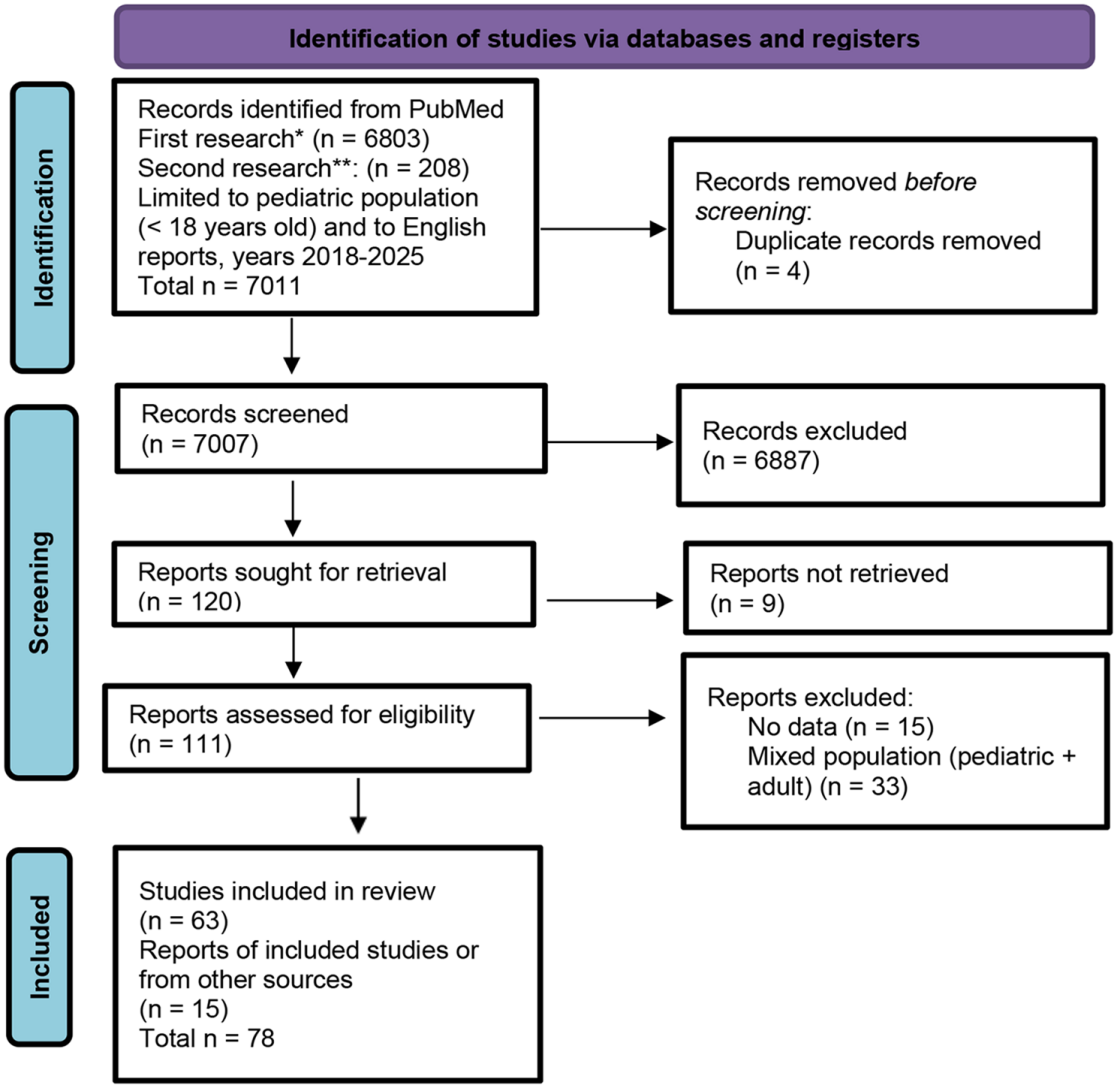
Flow chart of the selected process. *First research: Mesh terms: Smartphone OR Mobile Applications OR Internet Addiction Disorder OR Computers, Handheld OR Computers AND Behavior, Addictive OR Technology Addiction OR Health Risk Behaviors OR Risk Assessment OR Risk Factors. **Second Research: Mesh terms: media + device

From the first search, 6,803 records were identified; 82 met inclusion criteria after abstract screening. The second search yielded 208 articles, 33 of which were included. A final total of 63 studies were retained after full-text review, supplemented by 15 additional sources identified through cross-referencing. Each study was categorized by domain: obesity, cognitive development, sleep, ocular health, mental health, addiction/problematic use, violence/cyberbullying. Then, it was stratified by age group: children (< 13 years), adolescents (13–18 years), or mixed samples. Study quality and level of evidence were assessed through standard criteria, prioritizing systematic reviews, meta-analyses, randomized trials, and large-scale observational research. Extracted data included study design, country, population characteristics, exposure measures, and outcome indicators (Fig. 1).

## Results

**Fig. 2:**
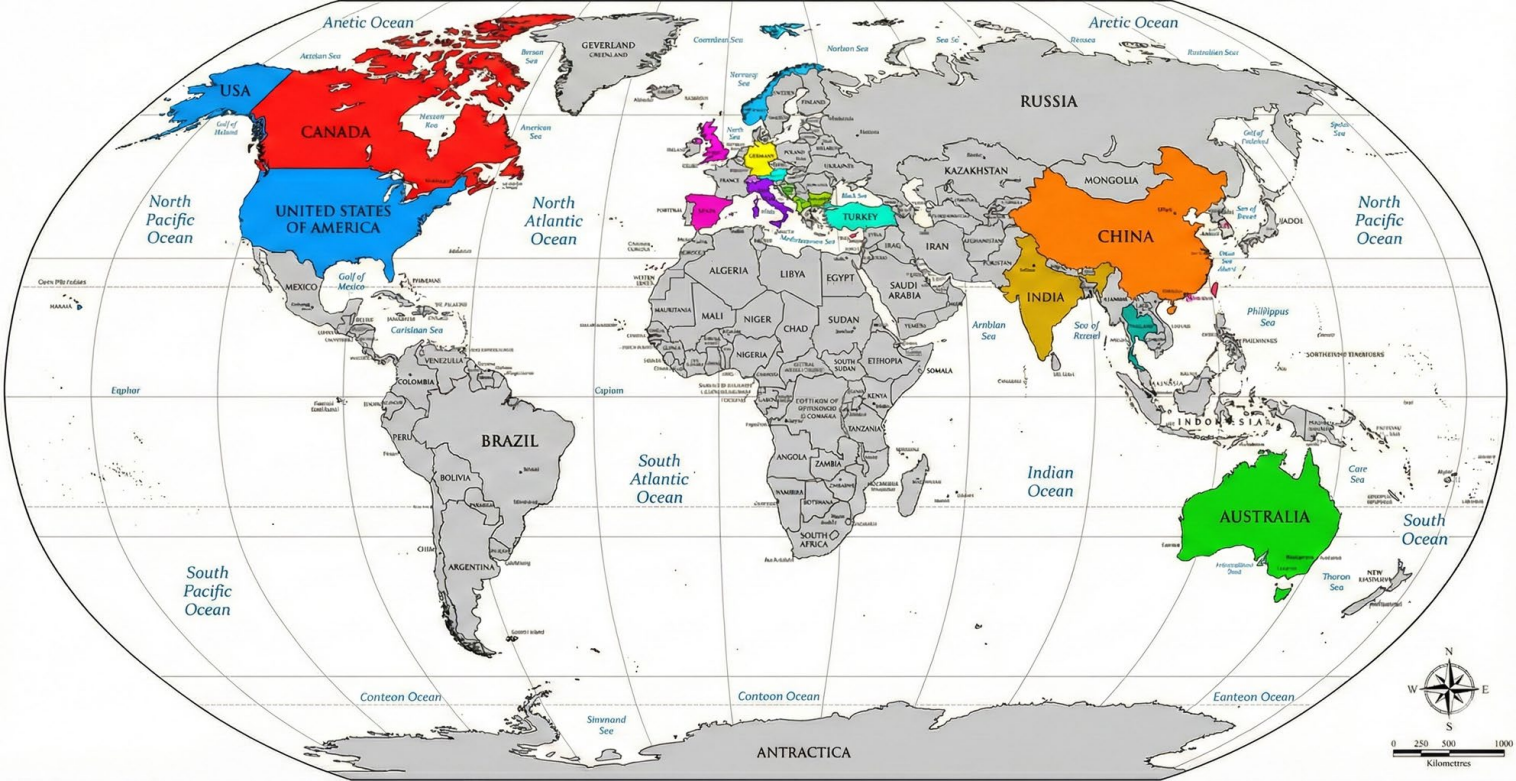
Country of provenience of studies included in the revision

The review was based on 78 articles and identified consistent evidence linking excessive or unsupervised digital exposure to adverse pediatric outcomes [1–78]. These findings are detailed by health domain below. The evidence tables compiled by the Commission summarize study typologies, populations, domains, and principal findings, supporting a comprehensive synthesis across health topics are available as supplementary material. Risk factors associated with increased screen time include child-related factors, such as age and gender, caregiver-related factors, such as parental screen time, maternal stress, parental attitudes and beliefs, socioeconomic and demographic factors, micro- and macro environment variables, such as access to devices, place of residence and season [38, 53, 59, 65]. The consistency of findings across different geographic and cultural contexts underscores the universality of this risk for the pediatric population (Fig. 2).

### Obesity

Childhood and adolescent obesity represent an increasingly prevalent global concern, with substantial implications for metabolic, cardiovascular, psychosocial, and developmental health. Out of the literature search, 18 studies were focused on obesity [2, 7, 9, 14–16, 18, 26, 27, 35, 39, 43, 45, 55–57, 60, 68]. Several mechanisms, such as displacement of physical activity, early establishment of unhealthy eating habits, and high sensitivity to persuasive food marketing, are particularly influential in this age group. In literature, prolonged daily screen time was associated with increased odds of being overweight and obesity, beginning in early childhood [16, 26, 40]. A growing body of evidence demonstrates that screen time plays a multifaceted role in the development of obesity. Mechanisms include: physical activity displacement as digital engagement replaces active play and reduces energy expenditure, food marketing and persuasive digital advertising since exposure to high-calorie food ads increases intake of sugar-rich, high-fat, and highly processed foods, distracted eating since consuming foods while viewing screens reduces satiety cues and increases caloric intake [2, 7, 14–16, 18, 26, 27, 35, 39, 45, 55–57, 68]. Moreover, a role is played by sleep disruption since evening screen use shortens sleep duration, modifies appetite-regulating hormones, and promotes weight gain and by family environmental factors considering that parental screen behaviours, household media saturation, and socioeconomic factors influence children’s screen exposure [9, 43]. Children exposed to screens for more than one hour daily exhibited significantly higher body mass indices (BMI) and lower levels of physical activity [43]. Furthermore, screen time beyond two hours per day increased risk by an additional 26%, confirming a graded effect. These findings are consistent with evidence from the USA, where screen exposure exceeding 1–1.5 h/day was associated with overweight in children across the 0–18 age spectrum, with particularly strong effects in younger cohorts [45]. Additional longitudinal evidence from Germany, involving over 1,700 primary schoolchildren with a mean age of approximately 7 years, found that screen time even slightly above one hour per day doubled the risk of becoming overweight or developing abdominal obesity within the following year [68]. These findings emphasize the powerful cumulative effect of screen-related sedentary behaviour on fat distribution starting in early childhood. Digital exposure promotes sedentary behavior, disrupts satiety cues during meals, and encourages unhealthy food consumption through targeted advertising. The contribution of sedentary digital behaviour to childhood adiposity is clearly illustrated in several studies. In a large clinical study involving Chinese preschoolers aged 3–6 years, time spent in sedentary screen activities predicted a higher likelihood of overweight. Specifically, each increment in screen exposure was associated with a significantly increased odds ratio for overweight status at preschool age [35]. Similarly, a systematic review conducted in the UK focused on children younger than 12 years found a robust association between the availability of electronic devices in the home environment and increased childhood adiposity, with 21 out of 29 included studies confirming this link [39]. Some studies provide insight into cognitive or behavioural correlates that may indirectly contribute to weight gain. For example, excessive screen time often co-occurs with dysregulated eating behaviours, such as consuming meals while watching television. A narrative review from Canada highlights that children who eat in front of screens exhibit reduced satiety, poorer diet quality, and higher caloric intake, contributing significantly to chronic weight gain [14]. Particularly in younger children, eating during screen use may disrupt the development of normal interoceptive cues, making them more vulnerable to overeating later in life. Furthermore, advergames and digital food advertisements significantly increased unhealthy food intake in pediatric participants [7]. While these findings encompass broad age ranges, experimental evidence suggests children are uniquely susceptible due to limited critical thinking about marketing content. A large systematic review covering 81,365 participants aged 7–18 years provides robust evidence linking internet addiction and obesity-related behaviours in either children and adolescents [18]. The mechanisms at the base of a strong association between internet addiction and increased BMI include: poor eating habits associated with excessive internet use, emotional eating, often triggered by stress or prolonged online engagement, disrupted weight-control behaviours, a documented positive correlation between internet and food addiction. Several studies confirm that adolescents exhibit significant weight gain associated with prolonged sedentary screen time. A scientific statement from the American Heart Association reported that adolescents who exceed two hours of screen time daily have a 1.8-fold higher risk of adiposity [15]. The association is stronger than in younger children, likely reflecting increased autonomy in leisure choices, greater use of social media, and reduced parental monitoring. Meta-analytic evidence also indicates that screen time ≥ 2 h/day is associated with a 67% increased risk of overweight/obesity among children and adolescents, with adolescents often showing the strongest effect sizes [26]. Adolescents are highly exposed to targeted digital food marketing, particularly through social media platforms where advertisements are embedded in influencer content, short videos, and interactive media. Exposure to energy-dense, nutrient-poor food marketing is associated with increased cravings, impulsive snacking, and preference for high-calorie foods [16]. Unlike younger children, adolescents often consume food during prolonged gaming or streaming sessions, reinforcing regular caloric intake during sedentary activities. This pattern leads to chronic positive energy balance and contributes to the rising prevalence of overweight in teenagers. Emerging research suggests that engagement with “advergames” and interactive food content may have even stronger effects in adolescents than in children, as adolescents demonstrate greater autonomy over food choices and access to discretionary spending [7]. These findings highlight that in adolescence, obesity risk may be driven not only by sedentary screen time but also by psychobehavioral dysregulation and compulsive digital use, which modify food intake patterns. Adolescents with higher levels of internet addiction exhibit more emotional eating behaviours, suggesting that digital media may indirectly influence obesity through psychological pathways [18]. Finally, a systematic review and meta-analysis from Iran involving 151,763 participants demonstrated that increased screen time is associated not only with obesity risk but also with higher hypertension risk across children and adolescents [27]. The review showed a dose-response relationship indicating that every additional 50–150 min of screen time substantially increases hypertension risk across age categories [60]. These findings reveal that obesity associated with screen exposure is part of a broader cardiometabolic risk spectrum, which becomes evident even in childhood.

### Cognitive development

Digital media exposure has become an integral component of childhood and adolescence, prompting extensive research into how screen-based activities affect cognitive growth, learning, attention, executive functioning, and academic outcomes [1, 2, 4, 5, 7, 21, 32, 36, 38, 41, 43, 53, 56, 59, 60, 62, 71]. An excessive or poorly mediated screen use has been linked to delayed language, impaired attention, and reduced executive functioning in younger children [43]. Among adolescents, the evidence points toward associations between heavy digital engagement, particularly social media, multitasking behaviour, and problematic internet use, and diminished concentration, increased attention deficit hyperactivity disorder (ADHD) like symptoms, and lower academic performance [60].

In early childhood, multiple studies underscore the developmental risks associated with excessive screen exposure during this sensitive period, particularly when screens displace high-quality caregiver interaction or occur without adult mediation [41]. A large Canadian study of 18-month-old children found that each additional 30 min of daily mobile device use was associated with more than double the odds of parent-reported expressive speech delay [71]. These findings align with earlier observational research that young children exposed to frequent digital media often show reduced verbal output and slower acquisition of vocabulary compared to peers with lower exposure or with greater levels of adult interaction during media use [41]. The mechanism appears partly attributable to decreased conversational turn-taking, as screen use tends to reduce opportunities for responsive verbal engagement between caregivers and children [41]. Additionally, the presence of background TV—even when children are not actively watching—disrupts play quality and verbal interaction, further limiting opportunities for language development [56]. Evidence indicates that early and unmonitored screen exposure can hinder the development of executive functioning, including working memory, response inhibition, and problem-solving skills [43]. Literature is consistent with association between screen time in early childhood and poorer fine motor and gross motor skills, as well as weaker self-regulation capacities [43]. A link between screen media and attention difficulties have been documented as early as toddlerhood. In Japan, prolonged TV viewing at 18 months predicted later hyperactivity, inattention, and social problems at age 30 months [56]. Similarly, U.S. data show that children who exceed two hours of daily screen use at age two exhibit greater difficulties in emotional self-regulation and behavior management [56]. These findings reinforce the hypothesis that early screen use can disrupt the development of attentional systems, particularly when media content is fast-paced or overstimulating [56]. Furthermore, children exposed to violent or high-intensity content may experience heightened arousal that interferes with settling, sustained attention, and impulse control [43]. Children exposed to narrative e-books or educational TV can benefit from well-structured content, but only when accompanied by adult co-viewing, which greatly enhances comprehension and vocabulary acquisition [56]. Co-viewing appears to triple word learning compared to solitary viewing, likely due to scaffolding and real-world contextualization offered by caregivers [53, 56]. In contrast, unmediated, passive consumption is associated with weaker comprehension and lower transfer of learned concepts to real-life contexts [41]. Neuroimaging evidence demonstrates measurable changes in brain structure related to heavy digital media use among preschoolers; higher ScreenQ scores, representing more intensive or higher-risk media behaviour, correlate with reduced cortical thickness and sulcal depth in regions linked to visual processing, attention regulation, memory encoding, and early literacy skills [4]. Such findings suggest potential alterations to neural networks foundational to early cognitive functioning, though causality remains under investigation [4].

Among adolescents, problematic gaming and frequent social media checking are associated with attentional deficits and lower academic performance [2, 62]. Several studies report that adolescents with problematic gaming or excessive digital involvement show structural brain differences in regions involved in cognitive control and attention [62]. Neuroimaging data describe reduced grey matter volume in prefrontal, temporoparietal, and subcortical regions among adolescents with problematic gaming behaviours, as well as decreased interhemispheric connectivity within the corpus callosum [62]. These structural alterations resemble patterns found in substance-based addictions and may contribute to difficulties in impulse regulation, working memory, and sustained attention [62]. Additional meta-analytic findings show small but consistent positive associations between screen time and ADHD-like symptoms in children and adolescents combined, suggesting that attentional vulnerabilities may be exacerbated by intensive media use [21]. Adolescents who frequently check social media throughout the day show significantly higher odds of exhibiting ADHD symptoms over a 24-month period, with the prevalence of symptoms rising in proportion to the number of high-frequency media behaviours reported [60]. The evidence underscores the critical importance of delaying unsupervised use and promoting adult-mediated, high-quality educational content [38, 53]. Screen use, particularly social networking, has correlated with poorer academic performance in multiple studies [2]. Adolescents often engage in multitasking—switching between studying, messaging, and entertainment—which is associated with fragmented attention and reduced learning retention [7]. Early and intensive smartphone acquisition also appears to impact academic trends [32].

### Sleep

Studies repeatedly demonstrate that screen exposure, especially in the evening or at night, interferes with sleep duration, sleep onset, circadian rhythm regulation, and sleep quality [1, 2, 6–9, 16, 22, 40, 42, 43, 48, 56]. These effects occur through multiple mechanisms, including time displacement, psychological stimulation, and physiological disruption of melatonin secretion due to blue-light exposure. Research on younger children consistently shows both direct and dose-dependent associations between screen exposure and sleep disturbances. Infants show significantly reduced sleep duration for each additional hour of screen use, indicating that vulnerability begins in the earliest stages of development [6]. Among infants and toddlers, increased screen use is further associated with longer sleep-onset latency, shorter night-time sleep, and greater daytime sleepiness, with nighttime exposure to screens exacerbating the severity of sleep deficits [6, 43, 48]. These early findings highlight that very young children have limited capacity to regulate arousal and are disproportionately affected by overstimulating or light-emitting devices. In children aged 3–5 years each additional hour of touchscreen has been associated to a sleep reduction of approximately 15 min [56]. Children who used devices for more than two hours per day had more than double the risk of sleeping less than recommended, and even one hour per day significantly impaired sleep, demonstrating a steep dose-response relationship [43]. Among school-age children (6–12 years), sleep disruption becomes increasingly linked to behavioural routines and the presence of electronic devices in the bedroom. Having screens in the bedroom or using devices immediately before bed is associated with later bedtimes, shorter sleep duration, and reduced sleep quality [48]. Every additional hour of video gaming in this age range results in approximately ten minutes of lost sleep, while evening television or smartphone use correlates with nocturnal awakenings and restless sleep patterns [48]. These cumulative impacts are significant: even small daily sleep losses accumulate into chronic sleep deficits that affect daytime cognition, mood, and academic performance. Mechanistic explanations for these patterns are well-established. More than 30% of preschoolers and most adolescents do not sleep the recommended hours, with screens contributing to delayed bedtime through time displacement, psychological stimulation, and melatonin suppression caused by blue-light exposure [8].

High-quality evidence confirms that screen use before bed is nearly universal among adolescents [8, 9, 22, 40, 48]. Literature reports that 72% of adolescents use cell phones in the hour before bed, 60% use computers or laptops, and 64% engage with music devices, with nearly one in five reporting being awakened by their phone multiple nights per week [22]. This nighttime connectivity is strongly associated with delayed sleep onset, shorter sleep duration, and poorer overall sleep efficiency [22]. These findings highlight that adolescents are not merely exposed to screens but often engage with them within the most vulnerable hours for circadian disruption. Large epidemiological surveys also link digital media use to insomnia symptoms in adolescents. A cross-sectional study of more than 7,500 German adolescents found strong associations between electronic media use and insomnia, with notable sex differences: computer and internet use were more strongly linked to insomnia in boys, while music listening showed stronger associations in girls [22]. Similarly, a Japanese study of more than 95,000 adolescents found that calling or texting after lights-out (reported by 8.3% and 17.6% of respondents, respectively) was independently associated with poor sleep, daytime sleepiness, and insomnia symptoms [22]. Of note, American teenagers who use screen devices for five or more hours per day have 80% higher odds of inadequate sleep compared to those with minimal use [9]. Digital engagement during nighttime hours not only delays sleep but also contributes to nocturnal awakenings, insomnia, and circadian rhythm disturbances. Hale suggests that most adolescents’ sleep deficits stem from the triad of time displacement, cognitive and emotional stimulation, and blue-light exposure, with digital multitasking intensifying cognitive arousal during nighttime hours [8].

### Visual health

Visual development in children and adolescents is strongly influenced by environmental conditions, including the intensity, duration, and proximity of near-work activities. Digital devices—particularly smartphones, tablets, and computers—have become major contributors to near-work exposure, and research increasingly identifies associations between screen use and a range of visual symptoms such as eye strain, reduced blinking, dry eye disease, photophobia, and myopia progression [1, 2, 16, 19, 30]. Evidence across age groups shows consistent patterns, with children exhibiting greater susceptibility to structural and refractive changes due to ongoing ocular development. Children experience pronounced ocular strain and refractive stress from prolonged screen exposure due to their shorter reading distances, immature visual system, and limited awareness of visual fatigue. Children may experience significant symptoms of digital eye strain, including headaches, blurred vision, and tired eyes, after prolonged screen use. Because children blink less frequently when focusing on screens, the ocular surface becomes more susceptible to tear film instability, leading to dryness and discomfort. Children with higher screen exposure are at higher risk of increased corneal epitheliopathy, dryness, and reduced tear-film stability, physiological factors that compromise visual comfort and may contribute to long-term ocular surface damage. Beyond ocular surface issues, blue-light exposure also influences visual health. While evidence does not support retinal damage from typical screen use, blue light can disrupt circadian rhythms and contribute indirectly to visual fatigue through delayed sleep onset and poor sleep quality, which in turn affects daytime visual comfort [8]. Additionally, the photopic environments created by screens increase visual contrast demands that may intensify accommodative stress in younger children. Myopia is one of the most significant long-term visual risks associated with digital media. Numerous studies identify prolonged near-work and reduced outdoor time as key factors [2]. Children who engage in more than two hours of screen time per day experience greater refractive shifts than those with lower use.

Adolescents commonly experience symptoms such as headache, blurred vision, and dry eyes, propelled by heavy near workloads, night time use, and sustained digital engagement following extensive use of social media, online gaming, and video streaming platforms [16]. Digital eye strain is particularly common in this age group because adolescents engage more heavily in sustained close-up viewing, often without taking breaks or adjusting lighting conditions. Excessive smartphone use is strongly associated with myopia progression among adolescents, providing evidence that the myopia risk extends beyond childhood and intensifies with heavier digital engagement. Adolescents also exhibit shorter blink intervals during smartphone use compared to reading print materials, increasing the likelihood of tear-film instability and evaporative dry eye. During the COVID-19 pandemic, remote schooling created unprecedented levels of continuous screen exposure among adolescents. A significant increase in eye strain, headaches, and difficulty focusing among secondary-school students, with symptoms correlating strongly with total daily screen time and reduced outdoor activities was noted. These findings highlight how prolonged digital demands in adolescence can overwhelm the visual system and contribute to persistent visual stress. Blue-light exposure can further disrupt visual comfort indirectly by affecting sleep, and therefore daytime visual performance. Blue light suppresses melatonin and delays sleep onset, indirectly contributing to tired eyes and impaired daytime visual stability [8]. This mechanism is particularly relevant to adolescents who often use devices late at night.

### Mental health

In younger children, mental health associations with digital media use largely reflect disruptions in social-emotional development, self-regulation, and early behavioral functioning. Research shows that high levels of screen use can displace crucial face-to-face interactions, impairing the development of emotional understanding and self-control [2, 3, 9, 16, 24, 31, 52, 54, 56, 58, 65, 69, 72, 78]. Caregiver use of screens to soothe infants and toddlers is associated with poorer social-emotional outcomes, including heightened emotional reactivity and weaker self-regulation [41]. When digital devices are regularly used as calming tools, children may miss opportunities to learn internal emotional regulation strategies—a foundational developmental skill. This displacement effect is particularly concerning under age three, when social-emotional circuits undergo rapid maturation. Problematic screen use in early childhood is associated with irritability, mood instability, and increased tantrums. Impulsivity, social withdrawal, and emotional difficulties occur more frequently among preschoolers who spend large amounts of time on digital devices, particularly when usage displaces peer interaction and exploratory play [1, 56].

Excessive screen use also correlates with attention-related symptoms that overlap with mental health challenges. Claussen found that children with higher screen exposure exhibited greater ADHD-like symptoms, such as impulsivity and emotional dysregulation, suggesting that attentional and emotional systems may be jointly affected by screen habits during early development [21]. Sleep disruption plays a compounding role in child mental health: sleep problems caused by evening or excessive screen use contribute to emotional instability, irritability, and mood disturbances during the day [6, 8]. This interaction underscores the interconnectedness of sleep and emotional well-being. Among school-age children, social media and digital gaming begin to take on greater psychological significance. While younger children use screens primarily for entertainment, older children increasingly encounter interactive digital environments. Children who spend excessive time gaming may exhibit irritability, social difficulties, and reduced interest in offline activities. These behavioral effects reflect an imbalance between digital and real-life social engagement.

Adolescents as well are particularly susceptible to the mental health effects of social media, online interactions, and digital multitasking. Depression and anxiety are two of the most widely documented mental health outcomes associated with adolescent digital media use. There is a clear association between excessive social networking and depressive symptoms, anxiety, and poor psychological well-being in adolescents, especially when usage leads to sleep loss or online social comparison [16, 24]. Systematic reviews consistently show that high social media engagement, particularly passive browsing, correlates with lower self-esteem, negative mood, and increased internalizing symptoms. Sleep disruption is another major mechanism driving mental health difficulties. Adolescents who use screens late into the night exhibit higher levels of depression, irritability, and emotional dysregulation due to shortened or fragmented sleep [8, 42]. Chronic sleep restriction amplifies psychological vulnerabilities and contributes to stress sensitivity, mood swings, and increased risk of internalizing disorders. Digital multitasking also affects adolescent mental health as adolescents frequently engage in multiple digital tasks concurrently, creating cognitive overload and associated stress [2]. High-frequency multitasking is linked to poorer mental health through increased anxiety, reduced attention span, and heightened feelings of being overwhelmed. Another key aspect of adolescent mental health relates to problematic digital behaviors. Schettler shows that problematic gaming and compulsive digital media use in adolescents are associated with impaired self-concept, heightened irritability, poor emotional regulation, and increased symptoms of anxiety and depression [62]. Social dynamics within digital environments play a central role. Adolescents who remain connected online at night—often out of fear of missing out (FOMO)—experience heightened stress, loneliness, and reduced emotional well-being [22]. Real-time online interactions, frequent notifications, and pressure to respond quickly create ongoing emotional stimulation that interferes with rest and mental recovery. Finally, cyberbullying constitutes another critical pathway linking digital media to adolescent mental health. Children and adolescents who experience online harassment show significantly higher rates of depression, anxiety, suicidal thoughts, and behavioral problems [16]. Because adolescents’ identities and peer relationships are fragile during this period, online rejection and social exclusion can have profound psychological impacts.

### Addiction and problematic use

Adolescents are particularly vulnerable to social media addiction, characterized by compulsive checking, inability to disengage, irritability when offline, and excessive preoccupation with online interactions [2, 11, 13, 16, 20, 23, 25, 29, 46, 47, 49, 50, 64, 66, 67, 70, 72–76]. Problematic smartphone use in adolescents is linked to dysfunctional metacognitive beliefs, such as using the smartphone to regulate emotions or cope with negative thoughts, which can reinforce addictive patterns [25]. These behaviours are reinforced by features such as infinite scroll, push notifications, and social reward loops [73].

Similarities between problematic smartphone use and behavioral addictions include loss of control, compulsive checking, and interference with daily functioning [25]. Risk factors for internet addiction include attending a high school class, poor academic performance, suffering from depression, coming from a disorganized family, having family members with Internet addiction, parents with a low level of education, restrictive parenting style. Protective factors include self-esteem, higher academic performance, positive qualities related to youth development, parents with a high level of education, social support [20, 23, 67, 70, 72, 75]. Adolescents who use devices late at night experience shorter sleep, which in turn increases emotional instability and reduces executive control, making them more prone to compulsive digital use [8, 22]. This creates a self-reinforcing cycle where emotional dysregulation fuels excessive use and excessive use worsens mental health. Gaming disorders have increased with the growing popularity of video games and online games. Approximately 1.5% to 9.9% of adolescents may have a gaming disorder. Gaming disorders are more common among young people due to free time and easy accessibility [23]. Adolescents with problematic gaming show neurobehavioral dysregulation with loss of control over gaming, withdrawal symptoms, tolerance-like escalation of time spent gaming, significant impairment in functioning. Neurobiological correlates, including altered activity in prefrontal and limbic regions involved in reward processing and executive control, have been found in adolescents with problematic gaming [23, 62]. Play-based prevention program can effectively reduce internet addiction risk in early adolescents [76]. Gamification-based educational interventions improve adolescents’ knowledge and attitudes toward preventing both substance and internet addiction. So, engaging, interactive learning approaches can play a meaningful role in reducing vulnerability to internet addiction and supporting healthier behavioral choices [66].

### Cyberbullying and online violence

Cyberbullying and online violence are included among the potential risk for minors involved in digital experience [2, 7, 12, 16, 17, 28, 33, 34, 37, 43, 44, 51, 61, 63, 77]. Cyberbullying represents a growing public health issue, with global prevalence rates between 20% and 33% among adolescents. Victimization triples the risk of suicidal ideation as deeply linked to depression, lower self-esteem, social withdrawal and anxiety [16]. Cyberbullying peaks during adolescence, when digital devices play a central role in social life and peer validation. Problematic digital use also increases the likelihood of perpetration. Adolescents who spend more time online, especially on unmonitored platforms, are more likely to engage in hostile messaging, rumor spreading, identity-based harassment and non-consensual image sharing [2, 7, 12, 16, 17, 28, 33, 43, 44, 51, 61, 63, 77]. These behaviours are magnified by online disinhibition, anonymity, and group dynamics, making cyberbullying a distinct and pervasive form of online violence [28, 63]. Nighttime engagement in social media not only disrupts sleep but also increases exposure to online conflicts and cyberbullying episodes [22]. Sleep disruption then heightens adolescents’ emotional vulnerability, which amplifies the psychological impact of victimization. Digital media use, including social networking and sexting, is linked to sexual coercion, abuse, and other forms of online interpersonal violence in young people’s relationships. The findings suggest that online platforms can amplify power imbalances and normalize harmful behaviors [17, 33]. Violent video games and social media in childhood/adolescence are a risk factor for aggressive attitudes in the short and long term. Watching violent programs during primary school correlates with aggressive attitudes in adulthood [33]. Viewing online pornography is associated with increased probability of the sending and receiving of sexual images and messages, known as sexting, sexual coercion and abuse, negative gender attitudes [63]. During the COVID-19 pandemic, while online platforms supported education and socialization, they also increased minors’ exposure to illegal networks, including paedophiles. This heightened online activity led to greater risks of grooming, exposure to suicidal content, and the distribution of child pornography, inflicting enduring psychological harm on victims [28].

Programs targeting online behavior can reduce cyberbullying involvement and improve youth mental health outcomes. Evidence demonstrate that cyberbullying and online violence are significant consequences of social media use, but that evidence-based interventions can mitigate harm and reduce psychological distress. Education on cyberbullying for adolescents, coping skills (stress management), empathy training, communication and social skills, and digital citizenship education, can get significant outcomes [34, 37].

## Discussion

The findings synthesized in this review demonstrate that digital media exposure represents one of the most influential and rapidly evolving determinants of paediatric health in the twenty-first century [1]. Across the domains explored, physical health, cognitive development, sleep regulation, ocular functioning, mental well-being, addiction vulnerability, and exposure to cyberviolence, a consistent picture emerges: the intensity, timing, quality, and context of digital engagement profoundly shape trajectories of childhood and adolescent development [26]. Without intentional guidance, environmental safeguards, and structured routines, these technologies can interfere with fundamental developmental processes [8]. The paediatric population, by virtue of ongoing neurological, emotional, and physical maturation, remains uniquely susceptible to the risks described throughout this document [19, 31]. So, the SIP Digital Dependency Commission underscores the urgent need for evidence-based, developmentally informed, family-centered strategies to mitigate these risks while promoting a balanced, healthy digital ecosystem for all children and adolescents.

One of the clearest insights emerging from the literature concerns the pervasive impact of excessive screen exposure on lifestyle patterns and metabolic health [13]. Across numerous studies, prolonged use of digital devices strongly correlates with sedentary behaviour, reduced physical activity, increased snacking, altered sleep rhythms, and heightened sensitivity to food marketing—all contributors to obesity and cardiometabolic dysregulation [2, 7, 9, 14–16, 18, 26, 27, 35, 39, 43, 45, 55–57, 60, 68]. Importantly, these associations are observed not only in adolescents but also in preschool-aged children, who exhibit increased odds of overweight status even with moderate daily exposure [51]. These findings emphasize that digital behaviours shape lifestyle routines long before children gain independence in mobility or food choices [7]. Digital media should be considered not merely as entertainment but as a potent behavioural environment influencing energy balance, food preferences, and physical activity from the earliest developmental stages [69]. Equally significant are the documented effects on cognitive development [1, 2, 4, 5, 7, 21, 32, 36, 41, 43, 56, 60, 62, 71]. In childhood, when neural networks governing language, executive function, attention, and socio-emotional regulation undergo rapid refinement, screen time and content may influence the growing [26]. Excessive or unmediated screen exposure, especially when it displaces caregiver interaction or free play, has been consistently associated with language delays, reduced expressive vocabulary, impaired attentional control, and poorer self-regulation [8]. Co-viewing with parents mitigates many of the risks identified [19]. These effects extend into school-age years and adolescence, where high screen use correlates with academic difficulties, multitasking-related attentional fragmentation, and neurobiological alterations in regions involved in executive functioning and reward processing [31]. Sleep health emerges as another critical domain negatively influenced by digital media, particularly in the evening hours [13]. A large and growing body of research across diverse countries shows that screen exposure delays bedtime, shortens sleep duration, increases night wakings, and disrupts circadian rhythms [12]. These effects begin in infancy, intensify in preschool and school-age children, and peak during adolescence, when nighttime use becomes nearly ubiquitous and social pressures to remain connected exacerbate sleep loss [51]. Sleep disruption, in turn, contributes to cognitive, emotional, and behavioural dysregulation, creating a reinforcing cycle of daytime fatigue, irritability, reduced academic performance, and increased reliance on digital media for passive relaxation [7]. The review also highlights substantial risks to visual health, particularly with the increasing use of smartphones and tablets that require sustained near-work at short viewing distances [69]. Symptoms such as dry eye, eye strain, headaches, and difficulties focusing are now observed frequently in paediatric patients and correlate directly with duration and intensity of screen use [1]. Of particular concern is the growing evidence linking prolonged near-work and reduced outdoor light exposure to myopia progression, a trend accelerating globally [26]. Because ocular structures continue developing throughout childhood and adolescence, excessive screen exposure during these periods may create long-term refractive consequences [8]. Mental health represents one of the most complex and concerning areas influenced by digital media exposure [2, 3, 9, 16, 24, 31, 52, 54, 56, 58, 69, 72, 78]. From early childhood through adolescence, excessive screen use correlates with emotional dysregulation, irritability, anxiety, depressive symptoms, impulsivity, loneliness, and increased psychological distress [31]. Social media platforms may introduce chronic pressures related to social comparison, body image concerns, and fear of missing out [13]. Such pressures are particularly potent during adolescence, a developmental period characterized by heightened sensitivity to peer evaluation and identity formation [12]. Closely related to mental health is the growing phenomenon of problematic digital use, including compulsive gaming, internet addiction, and dependency on social media [51]. Finally, cyberbullying and online violence further complicate the digital environment navigated by children and adolescents [7]. Victims face significantly higher risks of depression, anxiety, suicidal ideation, academic difficulties, and social withdrawal [69]. Perpetrators are also more likely to exhibit problematic digital use and mental health challenges [1].

Taken together, these findings illustrate that digital media exposure is a multifaceted determinant of child and adolescent well-being. Pediatricians should emphasize family-based lifestyle interventions, limit eating while using screens, and encourage outdoor activity. In conclusion, the Commission proposes the following evidence-based recommendations:

- Delay introduction of personal smartphones until at least 13 years of age; use simplified models until 18.
- Avoid unsupervised Internet access before age 13; enable parental controls thereafter.
- Postpone social media access ideally until 18, but not earlier than 14 years.
- Prohibit device use during meals, before bedtime, and within bedrooms.
- Encourage outdoor activities, sports, reading, and creative play as primary developmental experiences.
- Promote school-based digital literacy education, including privacy protection, cyber-ethics, and critical evaluation of sources.
- Pediatricians should routinely evaluate screen habits during visits, screen for risk factors (male sex, ADHD, anxiety, familial dependency), and counsel families using anticipatory guidance.

## Conclusion

After a systematic literary review, the SIP Digital Dependency Commission presents a set of comprehensive, evidence-based recommendations for families, pediatricians, educators, and policymakers These recommendations emphasize delaying personal smartphone ownership until at least age 13 and advocating for the use of simplified, less immersive devices until adulthood; avoiding unsupervised internet access before adolescence; postponing social media use ideally until age 18, with an absolute minimum of 14; prohibiting device use during meals, in bedrooms, and within the hour before bedtime; and prioritizing real-world interactions—outdoor play, sports, creative activities, reading, and family engagement—as the foundation of healthy development.

## Supplementary Information

Below is the link to the electronic supplementary material.

Supplementary Material 1

## Data Availability

Data and material are available at dr Bozzola’s office.

## Abbreviations

IGD: Internet Gaming Disorder
PSMU: Smartphone use
SIP: The Italian Society of Pediatrics
BMI: Body mass index
ADHD: Attention deficit hyperactivity disorder
FOMO: Fear of missing out
