# Supplementary material for: Digital media exposure and pediatric health: the recommendations from the Italian Society of Pediatrics Digital Dependency Commission
Source: Ital J Pediatr. 2026 Jan 23;52:30. doi: 10.1186/s13052-026-02198-6 (PMC12910747; doi:10.1186/s13052-026-02198-6)
Supplement: Supplementary file 1 — Supplementary Material 1 [file 13052_2026_2198_MOESM1_ESM.docx]

| **Reference (author, year)** | **Type of Publication** | **Country**  **(Continent)** | **Population age (y.o.)** | **Number of cases** | **Domains** | **Highlighted** |
| --- | --- | --- | --- | --- | --- | --- |
| Aglipay M., 2020 | Review Article | Canada | Children and adolescent | N.A | Obesity | The study investigates the underlying mechanisms for excessive weight correlating to media device use: displacement hypothesis (creen time replaces physical activity and sleep), food marketing (ads target foods high in sugar, salt, and fat), eating habits in front of screens, individual and family factors that influence exposure (children of parents who spend a lot of time on screens tend to imitate them; having devices in the bedroom increases usage; lower socioeconomic status is associated with more screen time and a greater likelihood of eating while watching). |
| Ariefdjohan M.,  2025 | Review  Article | USA | Children | N.A | Mental health | Research suggests an association between social media use and anxiety. Risk mechanisms include: self-esteem influenced by the number of "likes" and followers, social comparison (the tendency to present oneself positively creates false impressions of peers, lowering self-esteem and triggering social anxiety), and unrealistic beauty standards.  New anxiety-related phenomena associated with social media include: Nomophobia ("no mobile phone phobia"), with a prevalence ranging from 15.2% to 99.7%, fear of missing out, and cyberchondria (anxiety about serious illnesses, triggered by unverified online health information). |
| Barnett., 2018 | Review Article | USA | 0-18 years | N.A. | Obesity | The use of self-reported screen measures is associated with increased adiposity, with a 1.8-fold higher risk for adolescents who exceed 2 hours of screen time per day. Screen time is linked to adiposity in young people, with stronger evidence in cross-sectional studies than in longitudinal ones. Screen time, particularly television viewing, is also associated with adverse cardiometabolic outcomes. |
| Bozzola E  2018 | Review Article | Italy | Pre school children | N.A. | Cognitive development;  Sleep;  Visual health | Excessive use of media devices in pre-school children is associated with risks to language development, attention skills, and sleep regulation, especially when exposure begins early and is unsupervised. The Italian Pediatric Society highlights that early childhood is a critical period for brain development and learning through direct interaction, which screen use may displace. The authors recommend very limited, high-quality, and parent-mediated media use, prioritizing real-life play and caregiver interaction. |
| Bozzola E., 2019 | Review Article | Italy | Adolescent | N.A. | Obesity, cognitive development, sleep, visual health, mental health, addiction and problematic use,cyberbullying and online violence | Excessive media use during adolescence is associated with negative effects on attention, sleep quality, academic performance, and emotional regulation, particularly when screen time replaces physical activity and face-to-face interactions. The Italian Pediatric Society emphasizes that adolescence is a sensitive period for brain maturation, during which uncontrolled media exposure may interfere with executive functions and socio-emotional development. The authors recommend age-appropriate limits, parental supervision, and high-quality content to promote healthy cognitive and psychological development. |
| Bozzola E., 2022 | Review Article | Italy | <18 | N.A | Obesity; sleep; visual health; mental health; addiction and problematic use; cyberbulling and online violence | Media device use links to sedentary behavior and junk-food marketing, influencing eating habits, diet quality, and weight gain. Furthermore, it results in sleep deprivation due to delayed bedtimes and reduced total sleep duration and quality of rest in children. In adolescents, exposure to screen-based devices, online social networking sites, and video-sharing platforms is associated with sleep-onset difficulties, daytime sleepiness and fatigue, shorter sleep duration, later bedtime, and unfavorable changes in sleep habits over time. Minors can develop ocular disorders from excessive screen time, including myopia, eye fatigue, dryness, blurry vision, irritation, burning sensation, conjunctival injection, ocular redness, dry eye disease, decreased visual acuity, strain, fatigue acute acquired concomitant esotropia, and macular degeneration. Out of the 68 studies reviewed, depression was found in 27.9% of studies, general psychological problems in 20.6% (14 studies) and anxiety: 14.7% (10 studies). Risk of addiction is 14,7% over 10 studies In adolescence, social media intense or problematic use and frequent online contact with strangers are all independently associated with cyberbullying. Problematic social media use is an important driver of cyberbullying victimization and perpetration, especially among girls. The highest percentage is observed in adolescents, aged 13 to 15 years. Moreover, having daily access to the Internet and the sharing of gender on social media increased the likelihood of cyber victimization among adolescents aged 12–17 years. Those who use Tumblr and Snapchat were found to become victims even more frequently. There is a link among social media and sex related problems such as early sexual activity, exposure to pornography, and sexting. Especially for girls, higher social media use, associated with lower family affluence and poorer body image, are key to early sexual activity. There is a risk to me engaged in risky sexual behaviour, having unprotected sexual intercourse and sex with strangers with a odds of risky sexual behavior 1.23 higher in social media user than in other students. There is an association between exposure to media violence and aggressive behaviour, aggressive thoughts, and angry feelings. |
| Chang JH, 2019 | Clinical study | USA | 8-12 | 220 | Cyberbulling and on line violence | Exposure to violent video games increases children’s aggressive and dangerous behaviors, showing a causal link between violent digital media and real-world violence. |
| Che Mokhta M., 2025 | Review Article | Turkey | 7 -18 | 81365 | Obesity | There is a correlation between internet addiction, poor eating habits and emotional eating habits. |
| Chidi-Egboka NC., 2023 | Clinical Study | Australia | 6-15 years | 36 | Visual health | One hour smartphone use in school-aged children with healthy eyes results in decreased ocular comfort, slowing the blink rate to one third, with much longer open eye periods between blinks. |
| Chung et al., 2019 | Review Article | Hong Kong (Asia) | Adolescent | N.A. | Addiction and problematic use | Risk factors for Internet addiction include: male gender, attending a high school class, poor academic performance, suffering from depression, suicidal ideation, coming from a disorganized family, having family members with Internet addiction, parents with a low level of education, restrictive parenting style.  Protective factors include: self-esteem, higher academic performance, positive qualities related to youth development, parents with a high level of education. |
| Claussen AH, 2024 | Review Article | USA | Children and adolescent | N.A. | Cognitive development | Children and adolescents who spend more time on screens tend to show more Attention Deficit Hyperactivity Disorde-like symptoms (CC=0.07; 95% CI 0.02–0.12; k=9) and have higher odds of being classified in the Attention Deficit Hyperactivity Disorder group (OR=1.94; 95% CI 1.57–2.30; k=6). |
| de Zambotti M., 2018 | Review Article | USA | 10-18 years | N.A. | Sleep | A positive association between use of electronic media and insomnia complaints. Calling and texting after lights-out are independently associated with poor sleep, daytime sleepiness and insomnia symptoms. |
| Dixon D., 2023 | Review  Article | UK | 10-18 years | N.A. | Mental health | Parental technoference, or habitual disruptions in interpersonal relationships or time spent together caused by technological devices, is linked to adolescents' dependent cell phone use, depression, anxiety, and lower levels of life satisfaction. |
| Donati MA, 2025 | Clinical study | Italy | Adolescent | 93 | Addiction and problematic use | **Pr****oblematic smartphone use in adolescents is linked to dysfunctional metacognitive beliefs, such as using the smartphone to regulate emotions or cope with negative thoughts, which can reinforce addictive patterns.**  **Similarities between problematic smartphone use and behavioral addictions include loss of control, compulsive checking, and interference with daily functioning.** |
| Derevensk y et al., 2019 | Review Article | Canada | 0-18 years | N.A. | Addiction and problematic use | **I**nternet use disorder follows a trajectory like substance and gambling addictions. Prevalence estimates for internet use disorders range from 1.5% to 8.2%. Excessive internet use is associated with mental health problems and social difficulties.   - Between 0.2% and 12.3% of adolescents meet the criteria for gambling disorder, with 80% reporting having gambled at least once. Risk factors include being male, troubled family relationships, and mental health issues (anxiety, depression). - Gaming disorders have increased with the growing popularity of video games and online games. Approximately 1.5% to 9.9% of adolescents may have a gaming disorder. Gaming disorders are more common among young people due to free time and easy accessibility. |
| Fang et al., 2019 | Review Article | China | 0-18 | N.A. | Obesity | Screen time ≥2 hours per day is significantly associated with a 67% increased risk of overweight/obesity in children and adolescents compared to those who spend <2 hours per day. This positive association was consistent across different types of screen time (television and computer). |
| Farhani MA, 2023 | Review Article | Iran | Children and adolescent | 151763 | Obesity | Increased screen time is associated with higher systolic blood pressure (+ 1.9 mmHg (DMP 1.898; 95% CI 0.181–3.616; p=0.030; I²=83.4%) and with a higher risk of hypertension (OR 1.153; 95% CI 1.076–1.234; p<0.001; I²=83.2%).  There is a dose-response relationship: each 50, 100, and 150-minute increase in screen time is associated with a 17%, 38%, and 92% higher risk of hypertension in children; 8%, 17%, and 32% higher risk in adolescents. |
| Ferrara P, 2018 | Review Article | Italy | Children and adolescent | N.A. | Cyberbullying and on line violence | Online platforms allow harassment to occur continuously and publicly, intensifying its psychological and social impact on young people. Cyberbullying is a significant risk factor for psychological distress, somatic symptoms, and poor academic performance in children. Victims often show low self-esteem, depression, and behavioral issues, with some cases linked to suicidal behavior. Increasing adult awareness and pediatric training is crucial to support affected youth. |
| Ferrara P, 2020 | Editorial | Italy | Children and adolescent | N.A. | Cyberbullying and on line violence | During the COVID-19 pandemic, while online platforms supported education and socialization, they also increased minors’ exposure to illegal networks, including pedophiles. This heightened online activity led to greater risks of grooming, exposure to suicidal content, and the distribution of child pornography, inflicting enduring psychological harm on victims. |
| Fischer- Grote L., 2019 | **R**eview Article | **Au**stria | Children and adolescents | N.A. | Addiction and problematic use | Risk factors include using a smartphone for gaming and social networking, Female gender, harsh parenting style, low self-control  Protective factors include: cademic motivation and success |
| Fischer- Grote L., 2021 | Review Article | Austria | 10-18 | N.A. | Addiction and problematic use | Problematic smartphone use is linked to psychosocial health, quality of social life, school environment, emotions, self-perception, relationships with parents, financial resources. |
| Gajjar S, 2022 | Systematic Review | USA | Children and adolescent | N.A. | Visual health | near work activities, such as screen use at close viewing distances, are consistently associated with an increased risk of myopia development and progression in children and adolescents. |
| Gao YX, 2022 | Review Article | China | 8-18 | N.A. | Addiction and problematic use | Internet gaming disorder prevalence is 8.8% (95% CI 7.5–10.0). Higher baseline screen time predicts a higher risk of depression at follow-up (OR 1.20, 95% CI 1.12–1.28; I²=81%). There is an exposure threshold: >2 hours/day shows a stronger association (OR 1.55, 95% CI 1.31–1.84; I²=0%). |
| Gerosa T, 2023 | Clinical study | Italy | 10-18 years | 1672 | Cognitive development | The study reveales a negative effect of early smartphone acquisition on language proficiency trends for intensive screen media users |
| Hale L  2018 | Review Article | USA | Children and adolescent | N.A. | Sleep | The quality and quantity of sleep correlate to media device use due to time displacement (time on screens subtracted from sleep), psychological stimulation (which hinders relaxation), negative effects of blue light. More than 30% of preschoolers and the majority of adolescents do not sleep the recommended hours. Approcimately 75% of young people have at least one electronic device in their room and a significant part use it in the hour before sleep. |
| Hogan M, 2020 | Review Article | USA | Children and adolescent | N.A. | Cyberbulling and on line violence | Violent video games and social media in childhood/adolescence are a risk factor for aggressive attitudes in the short and long term.  Watching violent programs during primary school correlates with aggressive attitudes in adulthood. |
| Holly L .,2024 | Review Article | Switzerland | Children and adolescent | N.D. | Cyberbullying and on line violence | Digital environments, including social media platforms, act as key determinants of health by shaping social interactions, exposure to harm, and patterns of online behavior. The authors identify online violence and cyberbullying as public health concerns, emphasizing the need for population-level interventions to reduce digital harms and promote safer online social environments.Three groups of public health interventions that can help protect children from digital harm are delaying media and digital device use in young children:, reducing use among children of all ages and digital education for children and caregivers. |
| Hu R, 2021 | Clinical Study | China | 3-6 | 971 | Obesity | Children who spent more time in sedentary activities in front of a screen were more likely to be overweight [OR and 95% CI: 1.22 (1.03–1.45)]. |
| Huber B,  2018 | Clinical Study | Australia | 2-3 | 96 | Cognitive development, mental health | Children were more likely to delay gratification after playing an educational app than after viewing a cartoon (The odds for no touch were on average 8 times higher for the EduApp condition than for the Cartoon condition, 95% CI [1.69, 40.92], χ2(1) = 6.80, p = .009) |
| Hutson E., 2018 | Review Article | USA | 10-18 years | N.A | Cyberbullying and on line violence | This systematic review directly examines cyberbullying interventions and finds that programs targeting online behavior can reduce cyberbullying involvement and improve youth mental health outcomes. The findings demonstrate that cyberbullying and online violence are significant consequences of social media use, but that evidence-based interventions can mitigate harm and reduce psychological distress. Education on cyberbullying for adolescents, coping skills (stress management), empathy training, communication and social skills, and digital citizenship education, can get significant outcomes. |
| Hutton JS, 2022 | Clinical Study | USA | 3-5 | 52 | Cognitive development | There is an association between higher ScreenQ scores, a composite measure of digital media use, and lower cortical thickness and sulcal depth in brain areas supporting primary visual processing and higher-order functions such as top-down attention, complex memory encoding, letter recognition and social cognition |
| Kaur, 2019 | Review Article | India | 0-5 | N.A. | Cognitive development | Risk factors associated with increased screen time and cognitive development are:  -child-related factors: age (older children have more screen time), gender (being male is associated with more screen time and hyperactivity), behavioral traits (hyperactivity, inattention, sedentary activities).  -caregiver-related factors: parental screen time (parents who have high screen time themselves tend to have children with high screen time), maternal factors (maternal stress, being a first-time mother, and a shorter duration of breastfeeding), parental attitudes and beliefsarents' perceptions that digital media exposure is important or beneficial.  -socioeconomic and demographic factors: lower parental education, lower family income, and lower levels of parental physical activity.  - Environment: access to devices, home media rules (having a television switched on in the background, having a television on during dinner, and a lack of rules about screen time), residence and season (screen time tends to be higher in the winter). |
| Kininmonth AR., 2021 | Review Article | UK | <12 | N.A. | Obesity | Availability of electronic devices is associated with childhood adiposity in 21 out of 29 studies. The home media environment is associated with adiposity in childhood. |
| Krietsch KN., 2019 | Review Article | USA | 0-18 years | N.A. | Obesity, Sleep | Prolonged daily screen time was associated with increased odds of being overweight and obesity, beginning in early childhood. Studies consistently associated shorter and later sleep with greater sedentary and screen time. |
| Lawrence A,  2021 | Narrative review | USA | 0-5 | N.A | Cognitive development, mental health | An increased daily use of mobile devices is associated with an increased risk of delay in expressive language. Parental use of devices as a calming tool is also associated with lower social-emotional skills. |
| LeBourgeois MK., 2017 | Review Article | USA | 5-17 years | N.A. | Sleep | The study highlits a negative association between screen-based media consumption and sleep health, primarily via delayed bedtimes and reduced total sleep duration |
| Li C,. 2020 | Review Article | China | 0-7 | 50000 | Obesity; cognitive development; sleep; cyberbullying and online violence | In case of more than one hour of screen time per day, the risk of being overweight increases significantly (OR 1.872); with more than two hours per day the risk is 26% higher than for less exposed peers.  As for cognitive development, there is a rik for difficulty in both fine and gross motor skills, self-regulation and problem-solving skills, language delays and difficulties in math and reading  23 studies report a direct relationship between screen use and problems falling asleep, frequent awakenings and reduced rest hours. A meta-analysis shows that children who use devices have sleep disturbances. In case of one hour a day, sleep is impaired (OR 1,420). The risk is higher for more than two hours a day use (OR 2,283). |
| Li S., 2023 | Review Article | China | Primary school and middle school | N.A. | Cyberbulling and on line violence | Positive association between internet gaming disorder and aggression. Elevated aggression linked to excessive and problematic gaming increases the risk of online violence, including hostile communication, harassment, and cyberbullying behaviors in digital spaces. In details: primary school r = 0.400 (95% CI 0.363–0.434); middle school r = 0.292 (95% CI 0.236–0.347). |
| Lichtveld., 2018 | Review Article | USA | 0-18 years | N.A. | Obesity | Screen time exceeding 1 hour per day is associated with being overweight.  Watching television for more than 2 hours is associated with a higher risk of being overweight. |
| Lindenberg K, 2022 | Clinical study | Germany | 12-18 | 422 | Addiction and problematic use | This study conceptualizes gaming disorder and unspecified internet use disorder as behavioral addictions characterized by impaired control and functional impairment. The findings show that cognitive behavioral therapy –based preventive interventions significantly reduced symptoms of problematic and addictive digital use in adolescents, supporting it as an effective approach to addressing early addiction-related behaviors. |
| Liu J., 2024 | Review Article | USA | 0-18 | N.A. | Sleep | Increased screen media use is associated with longer sleep onset, shorter sleep duration, and increased daytime sleepiness; in infants, sleep duration decreased significantly with each additional hour of screen use. |
| Loleska S, 2021 | Review  Article | Macedonia | Children and adolescent | N.A. | Addiction and problematic use | The highest rates of problematic use or addiction are found when the first phone is obtained at an age under 13. |
| Lu Y, 2023 R | Review  Article | U.S.A. | 14-17 | 6032 | Cognitive development | Excessive screen use combined with insufficient sleep is linked to poorer academic performance and slower cognitive processing. The study emphasizes that balanced daily movement and lifestyle behaviors are crucial for supporting optimal cognitive development during adolescence. |
| Lund L  2021 | Review Article | Western Countries | 0-15 | 500371 | Sleep | In infants less than 5 years, television viewing and tablet use were linked to difficulty falling asleep and shorter overall sleep duration.  A more intense use of television seems to increase daytime naps, a sign of a lower consolidation of nighttime sleep. In those aged 6-12 years, evening use of television and smartphones is associated with nocturnal awakenings and restless sleep. Moreover, having electronic devices in the bedroom or using them before bed is associated with later bedtimes and shorter sleep duration. Video games and television are also related to delays in falling asleep: every hour a day of gaming translates into almost 10 minutes of sleep lost. Adolescents 13-15-year-old with intensive use of social media have a bad quality, with insomnia, frequent awakenings and rhythm disturbances. |
| Malinauskas R, 2019 | Review article | Asia and Europe | 11-18 years | N.A | Addiction and problematic use | Internet and smartphone addiction in adolescents share core addiction features such as compulsive use, tolerance, and withdrawal-like symptoms. The authors conclude that psychological interventions—particularly cognitive behavioral therapy -based approaches—are effective in reducing problematic and addictive technology use, although effect sizes vary depending on intervention intensity and duration. |
| Martin KB., 2021 | Review Article | Australia | Children and adolescent | 4656 | Addiction and problematic use | Excessive and unregulated screen time—often involving social media—negatively affects children’s well-being. Poor sleep and increased screen engagement are associated with heightened emotional dysregulation, which can increase vulnerability to cyberbullying involvement as both victims and perpetrators. |
| Nesi J., 2021 | Review Article | USA | Adolescent | N.A. | Cyberbullying and on line violence | Increased social media use is associated with higher exposure to online interpersonal stressors, including cyberbullying and online harassment. Such negative online interactions are identified as important mechanisms linking social media use to self-injurious thoughts and behaviors among adolescents. |
| Odgerss CL., 2020 | Review  Article | USA | Adolescent | N.A. | Mental health | Adolescents with personal or family vulnerabilities experience more negative experiences related to digital technology. |
| Oflu A, 2021 | Clinical Study | Turkey | 2-5 | 240 | Cognitive development | Screen time impacts on cognitive development. 98 out of 240 participants (40.8 %) had ≥ 4 hours of screen time. Caring by mother, age at first screen exposure 12 months, not co-viewing with parents were found to be associated with ≥ 4 hours of screen time. |
| Orben A., 2020 | Review  Article | UK | Adolescent | N.D | Mental health | A negative association between digital technology use and adolescent well-being is found. |
| Pan XF, 2021 | Review Article | China | 0-18 | N.A | Obesity | Overweight and obesity have increased rapidly in the past four decades. Am association with media device use is discussed. |
| Pardhan S, 2022 | Review Article | UK | Children and adolescents | N.A. | Obesity; sleep;  mental health | Increase screentime was noted in different countries during COVID19 pandemic.  Eating while watching television was positively associated with being overweight in subjects aged ≤18 years)  Screen device use of 5 or more hours/day among american teenagers resulted in 80% higher odds of inadequate sleep compared to controls (OR = 1.79, 95% CI: 1.54, 2.08)  Poorer mental health among children and adolescents who use screens for more than 2-3 hours a day and severe depressive symptoms associated with higher levels of screen time were noted. |
| Paulus FW., 2021 | Review Article | Germany | Children and adolescent | 300000 | Obesity; cognitive development; sleep; mental health; | Screen time is linked to unhealthy eating habits and an increased risk of obesity. One hour of television a day increased the risk of obesity by 10%, while three hours increased it by 27%. Eating in front of the television is associated with less healthy diets (higher carbohydrates and fewer vegetables).  In children aged 3–5 years, one hour of television a day reduces sleep duration, especially if the content is violent or seen in the evening. Each additional hour of touchscreen use reduces sleep by approximately 15 minutes.  Babies aged 4–35 months also show an increase in sleep disturbances proportional to the time spent in front of the television  Watching television for many hours at 18 months was linked to hyperactivity, inattention, and social problems at 30 months. Quality educational programs can help students learn new words, while others in children <2 years old are associated with less developed language. Co-viewing with adults can triple the words learned compared to watching alone. Conversely, the presence of television in the background reduces the quality of the game and hinders language development.  Media use exceeding 2 hours per day at 2 years of age was associated with greater difficulties in emotional self-regulation.  Exposure to background television from 6 months was associated with oppositional behavior, aggression, and emotional disturbances in 18 months old children. Prolonged television viewing at 18 months was linked to hyperactivity, inattention, and social problems at 30 months. |
| Perak AM., 2018 | Review Article | USA | 0-18 | N. A. | Obesity | To contrast overweight, moderate-to-vigorous intensity daily exercise is suggested as well as free time spent in front of a screen. In detalils,  at age 2–5 years, less than 1 hour/day and at age ≥6 years less than 2 hours/day screen time is suggested. |
| Piteo EM., 2020 | Review  Article | Australia | 5-18 | N.A. | Mental health | The study focuses on a positive correlation between problematic social media use and depressive symptoms in different countries. |
| Qi I.,  2023 | Review Article | China | 6-14 | 577251 | Cognitive Development | Prolonged screen time in school-aged children is associated with poorer attention, reduced executive functioning, and lower academic performance, particularly when screen use is passive (e.g., watching videos). The review also suggests that excessive screen exposure may interfere with language development and working memory, partly by displacing cognitively enriching activities such as reading, play, and social interaction. In contrast, moderate and educational screen use, especially when guided by adults, may support certain cognitive skills, highlighting the importance of content quality and balanced media habits. |
| Ra CK, 2018 | Clinical study | USA | 15-16 | 2587 | Cognitive development | High frequency of checking social media is associated with a significantly higher odds of having symptoms of Attention Deficit Hyperactivity Disorder across 24 months follow-up. students who reported no high frequency media use at baseline had a 4.6% mean rate of having Attention Deficit Hyperactivity Disorde symptoms at follow up vs 9.5% among those who reported 7 high-frequency activities and vs 10.5% among those students who reported 14high-frequency activities. |
| **S**anders T., 2024 | Review Article | Australia | 0-18 | 1 937 501 | Obesity; cognitive development; cyberbullying and online violence | Digital food advertising and advergames are associated with increased unhealthy food intake. Television use correlates to shorter sleep duration. Negative associations with learning/education are noted for television, video games, and general screen use. A positive effect is noted in case of co-viewing with a parent, educational television, and narrated e-books. Social media use shows a positive association with risky sexual behaviour: r = 0.21 (95% CI 0.14–0.28; k=14; N=23,096). |
| Santre S., 2023 | Review Article | Thailand | Adolescent | N.A. | Cyberbullying and on line violence | Constant connectivity and anonymity online facilitate repeated psychological aggression, making cyberbullying a distinct and pervasive form of online violence. Prevalence in adolescents varies widely (in 2011-2012, victims 14-17 years: Romania 37.3%, Greece 26.8%, Germany 24.3%, Poland: 21.5%; in 2014 in South Korea: 14.6% victims /6.3% perpetrators; Indonesia 12–13 years: up to ~80% report some victimization), with girls more often victims and boys more often perpetrators; heavier messaging/chat use increases risk. |
| Schettler L., 2022 | Review Article | Germany | Adolescent | N.A. | Cognitive development; mental health; addiction and Problematic Use | Magnetic resonance imaging revealed alterations in adolescents with problematic use, involving prefrontal, (for cognitive control functions), temporoparietal regions (for attention processes and self-concepts), as well as frontolimbic and subcortical regions (connected to emotion regulation and reward processing) compared with healthy controls. They differ in terms of reduced grey matter volume, reduced blood oxygen level, reduced fibre density within the corpus callosum and a reduced interhemispheric connectivity of prefrontal areas. |
| Stanley N, 2018 | Clinical Study | Bulgaria, Cyprus, England, Italy, and Norway | 14-17 | 4564 | Cyberbullying and on line violence | Digital media use, including social networking and sexting, is linked to sexual coercion, abuse, and other forms of online interpersonal violence in young people’s relationships. The findings suggest that online platforms can amplify power imbalances and normalize harmful behaviors. Viewing online pornography is associated with   increased probability of the sending and receiving of sexual images and messages, known as "sexting, sexual coercion and abuse, negative gender attitudes. |
| Stevens MW, 2021 | Review Article | Australia | Adolescent | N.A. | Addiction and problematic use | The prevalence of gaming disorder in adolescence is of 4.6%. Problematic gaming involves persistent and compulsive behavior leading to significant psychological, social, and academic impairment, underscoring its recognition as a public mental health concern. |
| Straker L., 2018 | Commentary | Australia | 0-8 | N.A | Mental health | Increased screen time and early exposure to digital technologies may affect children’s social and emotional development. Early and unregulated digital engagement may increase children’s vulnerability to negative online behaviors.. |
| Taghipour E, 2023 | Clinical study | Iran | 13-15 | 114 | Addiction and problematic use | Gamification-based educational interventions improve adolescents’ knowledge and attitudes toward preventing both substance and internet addiction. So, engaging, interactive learning approaches can play a meaningful role in reducing vulnerability to internet addiction and supporting healthier behavioral choices. |
| Theopilus Y, 2024 | SReview Article | Australia | <12 | N.A. | Addiction and problematic use | In 2022, the estimated global prevalence of Internet addiction in children was 13.82%. The high risk of Internet addiction in young children include limited self-control, incomplete brain development, poor parental regulation, and the influence of the child’s environment. The distal factors that contribute to determining the risk of Internet addiction in children under 12 years of age include family socioeconomic conditions, family dysfunctions (e.g., behavioral, academic, and social difficulties), and digital environments (e.g., types of devices used, online activities, and content accessed). The proximal factors include children’s, families’, and peers’ access to, behavior toward, and attitudes regarding Internet and media use. The maintaining factors include parent–child relationships, peer influence on Internet and media use, and children’s self-efficacy and self-regulation. |
| Throuvala MA., 2019 | Review  Article | UK | 11-17 years | N.A | Addiction and problematic use | Adolescent internet addiction is linked to poor self-regulation, emotional distress, and adverse academic and social outcomes. School-based preventive interventions targeting coping skills, emotional awareness, and healthy internet use are key to reducing addiction risk. |
| Traub M., 2018 | Research Article | Germany | 7.08 ± 0.6 | 1733 | Obesity | Excessive screen time was associated with a higher risk of being overweight and developing abdominal obesity at the one-year follow-up. Even spending more than one hour a day in screen time is enough to at least double the risk of becoming overweight or developing abdominal obesity. |
| Twenge JM., 2018 | Clinical study | USA | 2-17 | 40337 | Mental Health | After 1 hour of screen time per day, a further increased daily screen time is associated with poorer psychological well-being, including less curiosity, less self-control, greater distractibility, greater difficulty making friends, less emotional stability, being more difficult to care for, and an inability to complete tasks.   - Among 14- to 17-year-olds, heavy screen users (7+ hours/day) were more than twice likely to be diagnosed with depression (RR 2.39, 95% CI 1.54, 3.70), anxiety (RR 2.26, 95% CI 1.59, 3.22), receiving treatment from a mental health professional (RR 2.22, 95% CI 1.62, 3.03), or taking medication for a psychological or behavioral problem in the past 12 months (RR 2.99, 95% CI 1.94, 4.62). Even moderate screen use (4 hours/day) was associated with poorer psychological well-being compared to low user (less than 1 hour per day). |
| von Deneen KM., 2022 | Review Article | China | Adolescent | N.A. | Addiction and problematic use | 1.7–10.7% of adolescents meet the diagnostic criteria for Internet gaming disorder (IGD). Adolescents with IGD show alterations in frontostriatal circuitry similar to those observed in substance addiction, like nicotine addiction They have lower gray matter density in the left insula, left anterior cingulate cortex, left lingual gyrus, and left posterior cingulate cortex. |
| Van den Heuvel M., 2019 | Clinical Study | Canada | 1.5 | 893 | Cognitive development | Among children whose parents reported any mobile media device use (n= 200, 22.4%), the median daily mobile media device use was 15.7 minutes (range 1.4–300). For children who used a mobile media device, each additional 30-minute increase in daily mobile media device use was associated with increased odds of parent-reported expressive speech delay (ORa=2.33, 95% confidence interval, 1.25–4.82). |
| Vidal C 2025 | Review  Article | USA | **Children and Adolescent** | N.A. | Mental Health Addiction and problematic use | Social Media use is linked to depression and anxiety, suicidal thoughts and behaviours, poor sleep, body image, more distress, and poorer well-being.  In children, time spent on media, and especially problematic media use, is both associated with depression, most notably in females, and with younger onset age. Adolescents with attention-deficit hyperactivity disorder symptoms also present higher risk of problematic use than their peers. |
| Viros Martin CV., 2024 | Clinical study | Spain | 12-18 years | 737 | Mental Health Addiction and problematic use | - Adolescents, especially girls, spend substantial time on TikTok and show gendered patterns in the types of content they consume, most report a generally positive self-perceived digital well-being on the platform. However, greater daily use is linked to reduced agency in setting limits on time spent, and specific content types showed small positive correlations with aspects of digital well-being, suggesting nuanced relationships between how teens use TikTok and how they perceive its impact. |
| Yumru Menteş H., 2025 | Clinical study | Turkey | 11-12 | 90 | Addiction and problematic use | - A nurse-led, play-based prevention program can effectively reduce internet addiction risk in early adolescents. The results indicate improvements in self-control, awareness of healthy internet use, and reduced problematic online behaviors, with a significant reduction in the severity of addiction (IAT: 64.15 → 50.33; p < 0.001; large effect d = 1.178), in the average daily Internet usage time (2.53 h/day → 1.83 h/day; p < 0.001; moderate effect d = 0.688), and in the duration of digital gaming (1.57 h/day → 0.99 h/day; p < 0.001; large effect d = 0.978) in the experimental group. |
| Zhu C, 2021 | Review Article | China | Children and adolescent | N.A. | Cyberbullying and on line violence | From 2015 to 2019 the average global cyberbullying perpetration rate of 25.03%, average victimization was 33.08%. Verbal violence was the most common type of cyberbullying (victimization prevalence: between 5 and 47.5%; perpetration prevalence: between 3.2 and 26.1%). Visual violence was characterized by victimization ranging from 2.6 to 12.1%, and perpetration prevalence ranging from 1.7 to 6%. Risk factors include females and adolescents with mental health problems, the youth living in urban areas. Protector factors include parent-child relationships and active communication. |
| Zink J., 2020 | Review  Article | USA | 5-18 | N.A. | Mental health | - The type of screen may impac on the intensity of depressive symptoms related to screen use. Television viewing is less likely to be associated with depressive symptoms than computer use and video games. Passive social media use can be harmful because it increases a person's feelings of inferiority through social comparison. |
| Wan X, 2022 | Review Article | **C**hina | Middle-school students | 7897 | Addiction and problematic use | Mobile phone addiction is a a form of problematic internet-related behavior associated with reduced perceived social support among Chinese adolescents. The findings suggest that lower family and peer support increases vulnerability to addictive patterns of internet and smartphone use. Social support is a protective factors: greater social support corresponded to less cell phone addiction. (r =−0.121; 95% CI −0.188 to −0.053). |
